# Supplementary material for: PathIntegrate: Multivariate modelling approaches for pathway-based multi-omics data integration
Source: PLoS Comput Biol. 2024 Mar 25;20(3):e1011814. doi: 10.1371/journal.pcbi.1011814 (PMC10994553; doi:10.1371/journal.pcbi.1011814)
Supplement: S1 Supporting Information — File containing supporting figures and tables. Fig A in S1 Supporting Information: Fold changes in COVID-19 multi-omics data based on outcome (mild vs. severe cases). Fig B in S1 Supporting Information: Fold changes in COPDgene multi-omics data based on either COPD status or gender outcomes. Fig C in S1 Supporting Information: Pathway transformation enhances sensitivity to low signal-to-noise signals (COPDgene semi synthetic data). Y axis shows proportion of MWU tests significant at Bonferroni p ≤ 0.05, performed either on the pathway-level data or the molecular level data, at varying effect sizes shown on X-axis. Fig D in S1 Supporting Information: Violin plots showing log10 pathway size for KEGG and Reactome human databases, both for the original databases as well as the database specific coverage (COPDgene and COVID-19). Pathways used are Reactome and KEGG human multi-omics pathways, containing both metabolites and proteins. Fig E in S1 Supporting Information. Comparison of PathIntegrate methods classification performance using KEGG and Reactome pathway databases as well as molecular-level model based on semi-synthetic COPDgene data. Fig F in S1 Supporting Information. Comparison of PathIntegrate and DIABLO full/sparse models ability to correctly recall target enriched pathway based on semi-synthetic COPDGene data. ‘DIABLO pathway (loading)’ uses an RGCCA model with no regularisation, whereas ‘DIABLO pathway (sparse loading)’ uses an RGCCA model with L1 penalty. Fig G in S1 Supporting Information: Investigation of effect of sample size in PathIntegrate Single-View (PLS) classification performance on COPDgene data. Fig H in S1 Supporting Information: Performance of PathIntegrate and DIABLO vs. effect size, based on semi-synthetic data measured by AUROC. COVID-19 metabolomics and proteomics data were integrated in each model. A. Ability to correctly predict sample outcomes (case vs. control). We compared PathIntegrate Multi-View and Single-View to DIABLO using both [file pcbi.1011814.s001.pdf]

S1 Supporting Information

Authors:

Cecilia Wieder[1], Juliette Cooke[2], Clement Frainay[2], Nathalie Poupin[2], Russell Bowler[3], Fabien Jourdan[4], Katerina J. Kechris[5], Rachel PJ Lai[6], Timothy Ebbels*[1]

Affiliations:

[1]Section of Bioinformatics, Division of Systems Medicine, Department of Metabolism, Digestion, and Reproduction, Faculty of Medicine, Imperial College London, London, United Kingdom

[2]Toxalim (Research Centre in Food Toxicology), Université de Toulouse, INRAE, ENVT, INP-Purpan, UPS, Toulouse, France

[3]National Jewish Health, 1400 Jackson Street, Denver, CO, 80206, USA

[4]MetaboHUB-Metatoul, National Infrastructure of Metabolomics and Fluxomics, Toulouse, France

[5]Department of Biostatistics and Informatics, Colorado School of Public Health, University of Colorado Anschutz Medical Campus, Aurora, CO, United States of America

[6]Department of Infectious Disease, Faculty of Medicine, Imperial College London, London, United Kingdom

Related work

DIABLO [1] is a supervised method for multi-omics data integration based on generalised canonical correlation analysis (GCCA). It uses singular value decomposition to find a lower-dimensional representation of multiple omics input matrices and selects correlated variables which are associated with the phenotype of interest. It requires the user to specify a design matrix, representing the expected correlation between omics datasets in the model. The inputs for DIABLO are scaled N-by-M omics data matrices, rendering it also compatible with pathway-transformed data matrices.

MOGSA [2] is an unsupervised method for multi-omics data integration, designed to output a matrix of multi-omics single-sample pathway scores. It begins by integrating the data at the molecular level using multiple-factor analysis, followed by projecting a binary matrix of pathway-membership information onto the observations in the latent space, and finally multiplying together the latent space matrices of samples and pathways to produce an N-by-P pathway score matrix. The final pathway score matrix can be decomposed to investigate the contribution of each omics dataset. MOGSA, unlike PathIntegrate and DIABLO, is not a predictive model but rather a method for generating multi-omics pathway scores, which could be used as input to predictive models like PathIntegrate.

Like MOGSA, Multi-Omics Pathway Analysis (MOPA) [3] generates pathway-score matrices using non-negative tensor decomposition. It is designed for gene-based omics data such as mRNA, methylation, and miRNA data. MOPA uses a two-step process for generating pathway scores, firstly it employs a non-negative tensor decomposition to perform feature selection to find genes significantly associated with a phenotype, and secondly computes pathway scores using these genes with a method similar to Gene Set Variation Analysis [4]. Like MOGSA, MOPA allows the calculation of an 'omics contribution rate', to understand how different omics contribute to pathway score calculation.

Multi-Omics Factor Analysis (MOFA) [5] is an unsupervised latent-variable method for multi-omics data integration. It uses group factor analysis to decompose multiple omics matrices into loadings and score matrices, which can be sparse. MOFA could be used with ssPA score matrices as input, to form an unsupervised pathway-based multi-omics integration model. Similar to PathIntegrate, users can extract variable importances for each latent factor and the contribution of each omics to each factor.

Lilikoi 2.0 [6] is a metabolomics-specific pathway-based deep learning model. It uses Pathifier [7] to produce ssPA scores which are then input to a deep neural network, or other classifiers such as random forest or logistic regression. It offers prognosis prediction using a Cox proportional hazards model, as well as network-based pathway visualisation options for downstream analysis.

PathwayPCA [8] is a toolkit offering multiple pathway-analysis based utilities: 1) testing pathway association with an outcome (similar to conventional pathway analysis), 2) extracting important genes within a pathway using sparse modelling, 3) compute pathway scoring on important genes, which can be used as input for multi-omics analysis. The pathway scores are computed using Adaptive, Elastic-net, Sparse PCA) or Supervised PCA

(SuperPCA), introduced by the same authors. Similar to Lilikoi, the pathway-transformed output can be input to various downstream analysis such as survival analysis.

Integrative directed random walk-based method utilizing pathway information (iDRW) [9,10] is a method for generating ssPA scores based on utilising gene-gene topological interactions within pathways. Combining a gene-gene directed graph based on KEGG pathways and a random walk algorithm, iDRW was used to integrate gene-expression and copy number alteration data, resulting in a pathway score matrix. The authors demonstrated using iDRW scores improved survival prediction compared to molecular-level data as well as other ssPA scoring approaches.

Finally, we refer the interested reader to a comprehensive review by Maghsoudi et al. [11] which provides a systematic evaluation of 32 integrative pathway analysis methods. While the aforementioned methods all provide useful functionality for either multi-omics integration at the molecular level (DIABLO, MOFA), or the generation of pathway scores at either the single omics level (Lilikoi, PathwayPCA), or the multi-omics level (MOGSA, MOPA, iDRW), none of these provide a framework for pathway-based multi-omics data integration. PathIntegrate seeks to fill this gap, providing a user-friendly Python implementation of the Multi-View and Single-View frameworks which a) generate multi-omics pathway scores (based on the user's choice of ssPA methods), and b) apply state-of-the-art predictive models to identify perturbed pathways. Furthermore, the majority of methods for generating multi-omics pathway scores are not designed to incorporate metabolomics data and are primarily based on gene/protein identifiers. PathIntegrate is specifically designed for (but not limited to) the integration of metabolomics data alongside other omics, providing multi-omics pathways containing gene (ENSEMBL), protein (UniProt), and metabolite (ChEBI) identifiers. Finally, to enhance ease-of-use and seamless integration with other pipelines, PathIntegrate models are compatible SciKit-Learn estimators, enabling the use of various predictive models and parameter optimisation functions available in the SciKit-Learn library.

Supplementary figures and tables

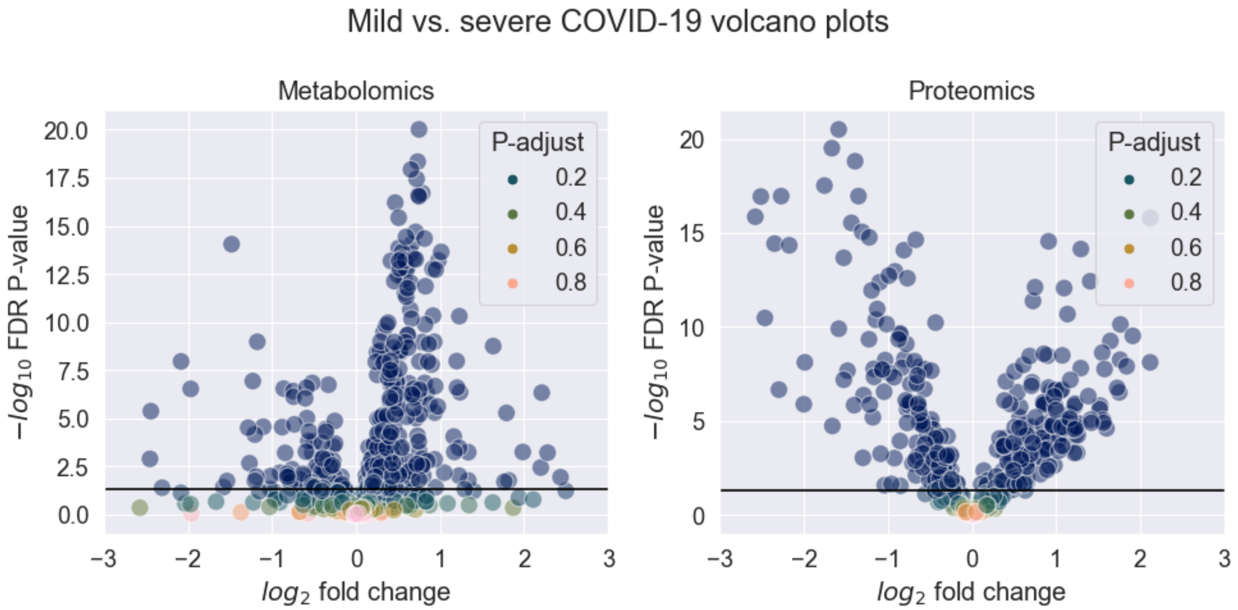

Fig A in S1 Supporting Information: *Fold changes in COVID-19 multi-omics data based on outcome (mild vs. severe cases).*

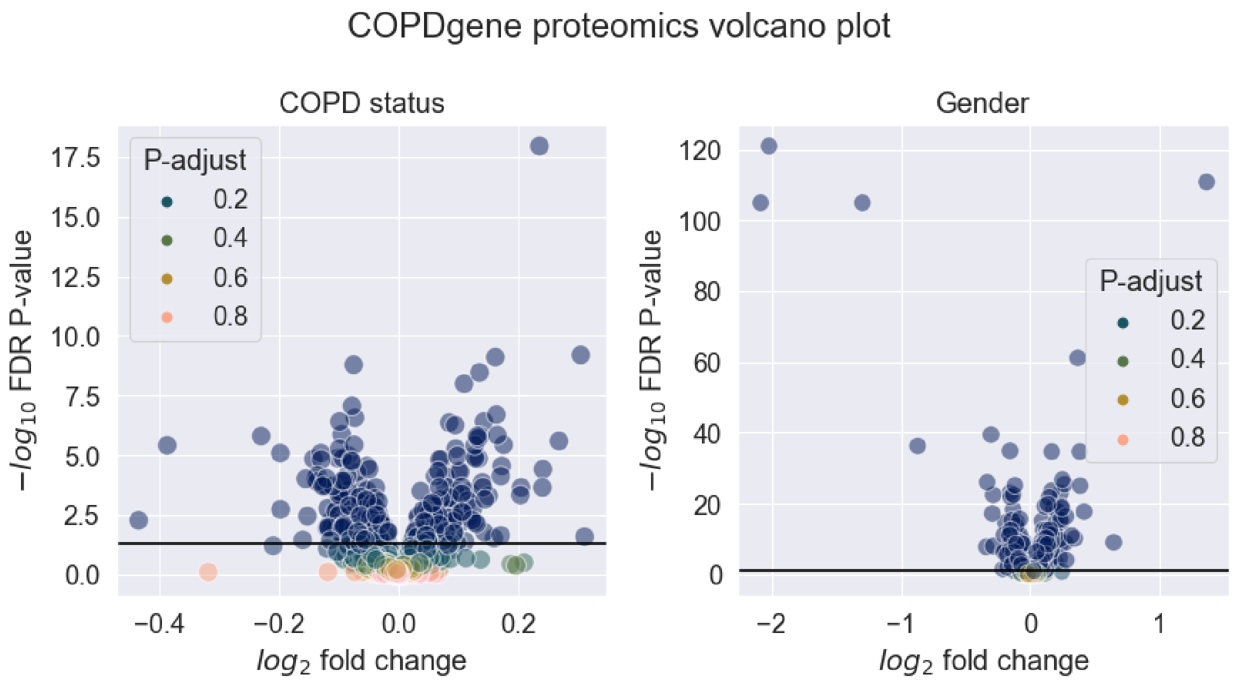
COPDgene proteomics volcano plot
COPD status
Gender
P-adjust
0.2
0.4
0.6
0.8
$-log_{10}$ FDR P-value
log_2 fold change

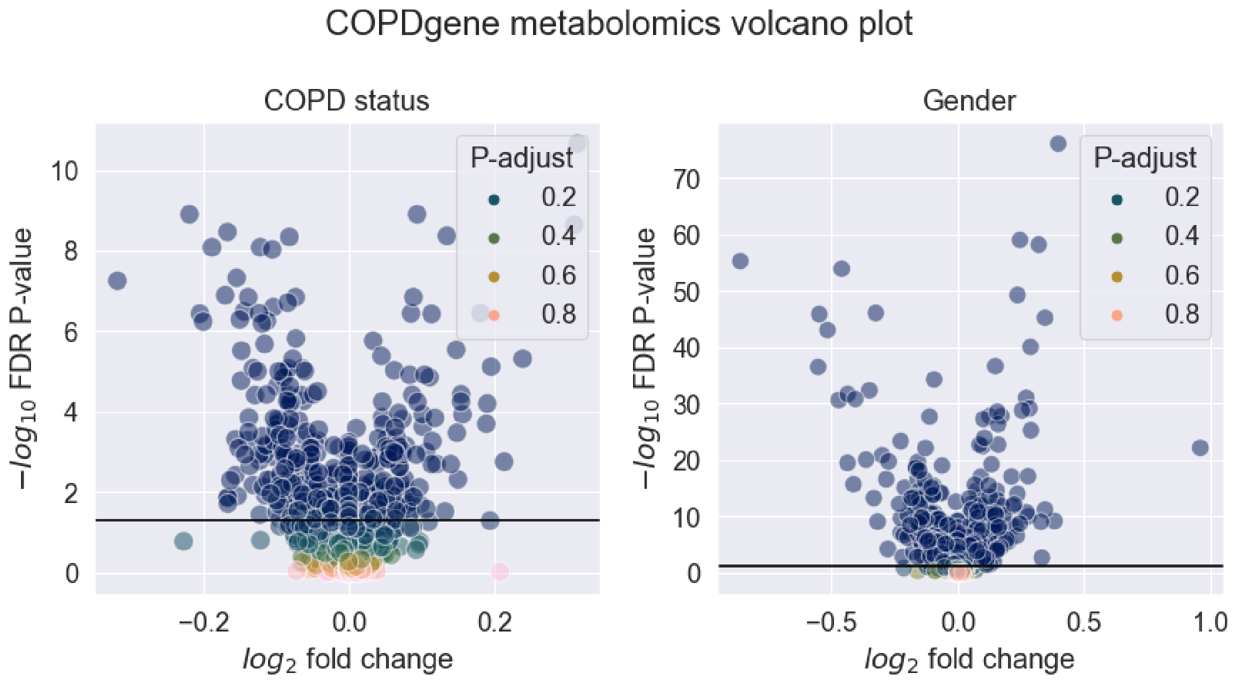
COPDgene metabolomics volcano plot
COPD status
Gender
P-adjust
0.2
0.4
0.6
0.8
$-log_{10}$ FDR P-value
log_2 fold change

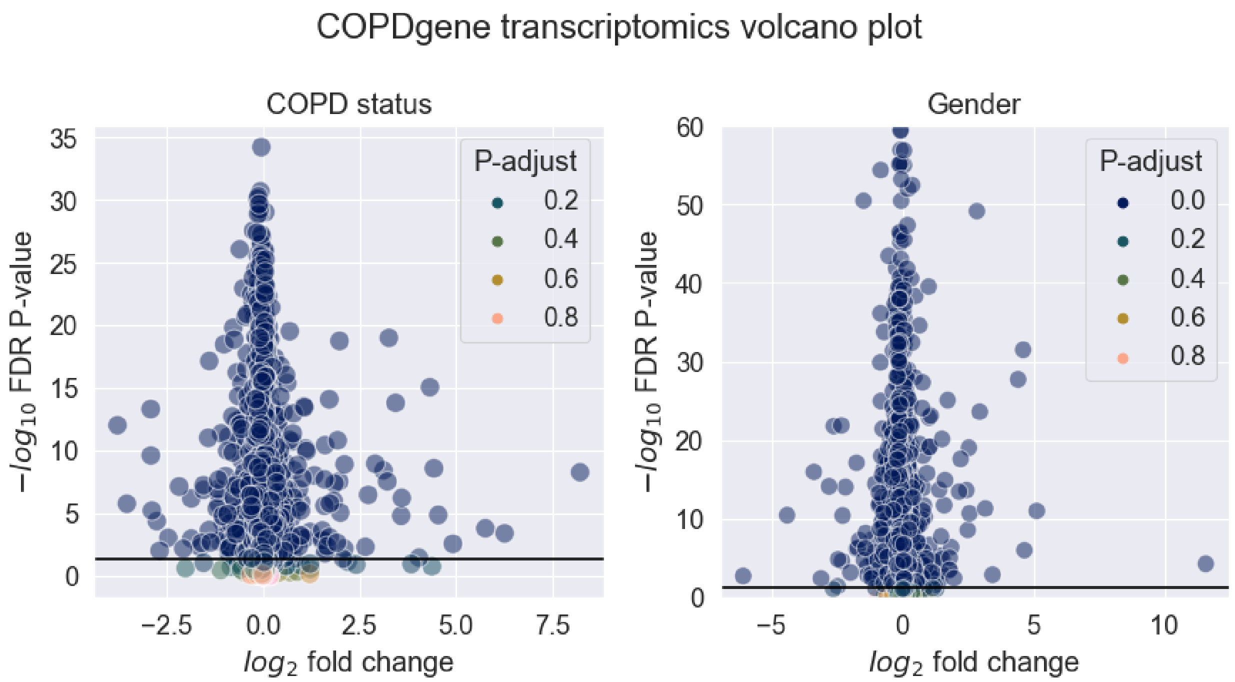

Fig B in S1 Supporting Information: *Fold changes in COPDgene multi-omics data based on either COPD status or gender outcomes.*

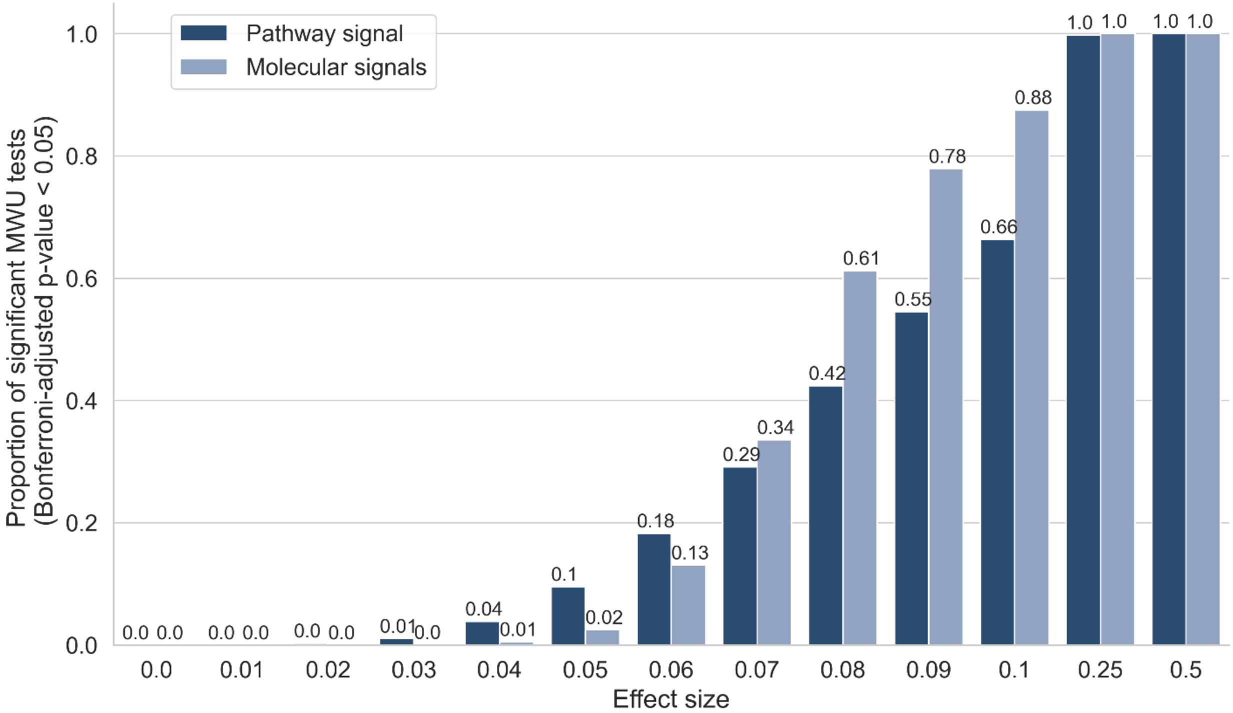

Fig C in S1 Supporting Information: Pathway transformation enhances sensitivity to low signal-to-noise signals (COPDgene semi synthetic data). *Y axis shows proportion of MWU tests significant at Bonferroni* $p \leq 0.05$*, performed either on the pathway-level data or the molecular level data, at varying effect sizes shown on X-axis.*

Pathway database influences model performance

The performance of pathway-based models is strongly dependent on the pathway definitions used. The number and composition of pathways varies between databases, and factors such as size, level of overlap, ratio of compounds to proteins/genes, etc. can all impact the models. We investigated the size of pathways in the Reactome human versus the KEGG human multi-omics pathway databases (those used in this work, where pathways can contain a combination of metabolites, proteins, and genes), and found KEGG to contain on average larger pathways (median size 96 molecules) than Reactome (median size 24 molecules). Reactome however contains more pathways (2,583) than KEGG (352). Importantly, these pathway database statistics are influenced by the molecules profiled in the dataset at hand, as only molecules that map to pathway identifiers will be included in the modelling. We investigated the pathway size distribution in two datasets, COPDgene and COVID-19 and found that the general trend was the same: KEGG pathways are generally larger than Reactome pathways (*Fig D in S1 Supporting Information*).

We also investigated the pathway annotation levels of genes, proteins, and metabolites, i.e. the percentage of molecules profiled in a dataset with a valid pathway database identifier (ENSEMBL, Uniprot, or ChEBI) assigned to pathways. Although results are highly dataset and assay-dependent, when considering the COPDgene and COVID-19 datasets and the Reactome pathway database (*Table A in S1 Supporting Information*), we found proteomics data to have the highest percentage of total molecules profiled mapping to pathways (>70% for both datasets). Metabolomics data had the lowest percentage of molecules mapping to pathways (16.9% for COPDgene and 23.9% for COVID-19). This is likely due to the specificity of the ChEBI identifiers, particularly for chemical subclasses such as fatty acids, where molecules i.e. lipids can be annotated to a very high level of specificity depending on side chain composition etc, but these are not yet annotated to pathway databases at such a high level of specificity. Bulk transcriptomics data was not available for the COVID-19 data, but in the COPDgene dataset only 27% of ENSEMBL genes mapped to Reactome pathways, demonstrating that the annotation issue is not specific only to metabolomics data, but can also affect sequencing-based omics such as transcriptomics, where thousands of genes are yet to be added to pathways.

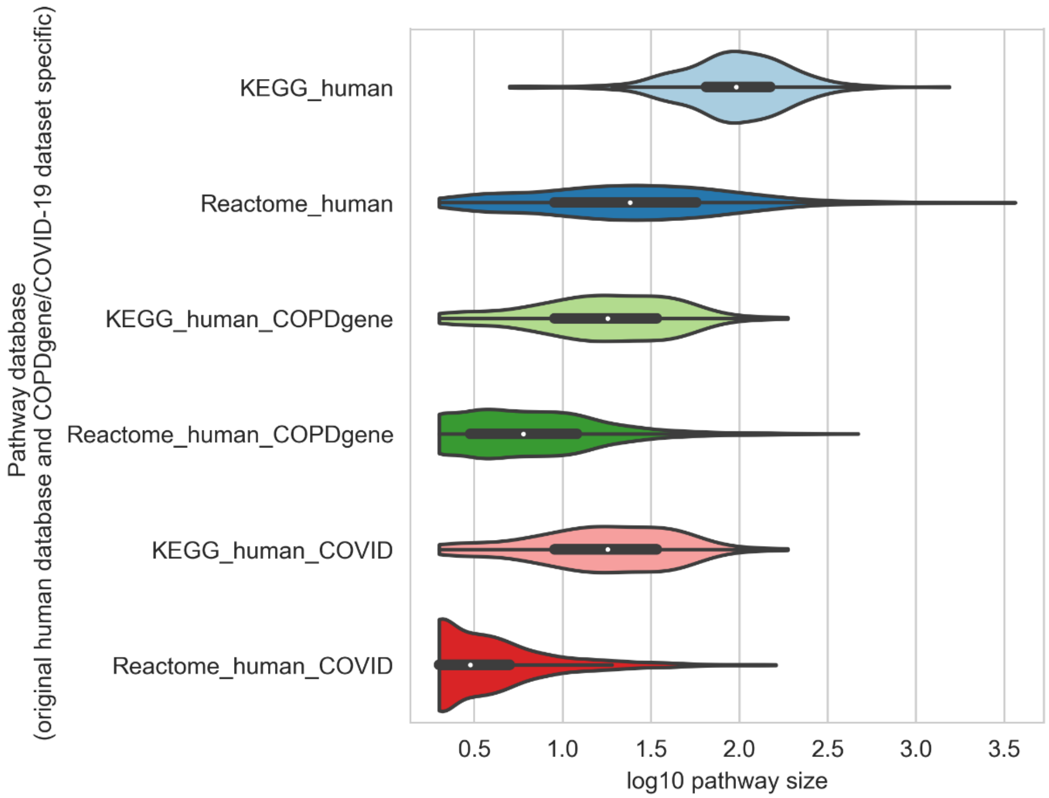

Fig D in S1 Supporting Information: *Violin plots showing log10 pathway size for KEGG and Reactome human databases, both for the original databases as well as the database specific coverage (COPDgene and COVID-19). Pathways used are Reactome and KEGG human multi-omics pathways, containing both metabolites and proteins.*

Table A in S1 Supporting Information*: Percentage of molecules with a valid identifier (ChEBI, UniProt, or ENSEMBL) in single omics mapping to Reactome human pathways. A lower percentage of molecules mapping to pathways means a greater percentage of molecules do not yet map to pathways and are not incorporated into pathway-based analyses.*

	**% of molecules with an identifier mapping to pathways**		
**Dataset**	**Metabolomics (ChEBI)**	**Proteomics (UniProt)**	**Transcriptomics (ENSEMBL)**
**COPDgene**	16.9	81.5	27.5
**COVID-19**	23.9	77.9	NA (No transcriptomics data)

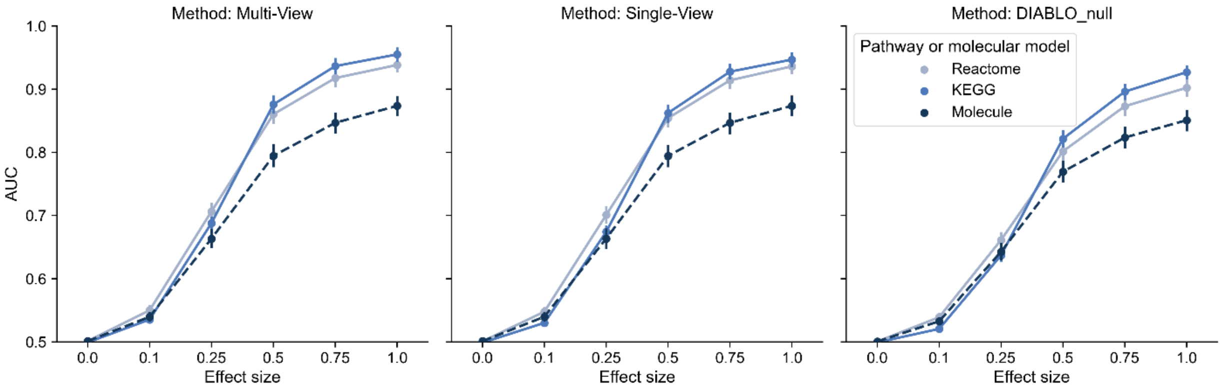

Fig E in S1 Supporting Information. *Comparison of PathIntegrate methods classification performance using KEGG and Reactome pathway databases as well as molecular-level model based on semi-synthetic COPDgene data.*

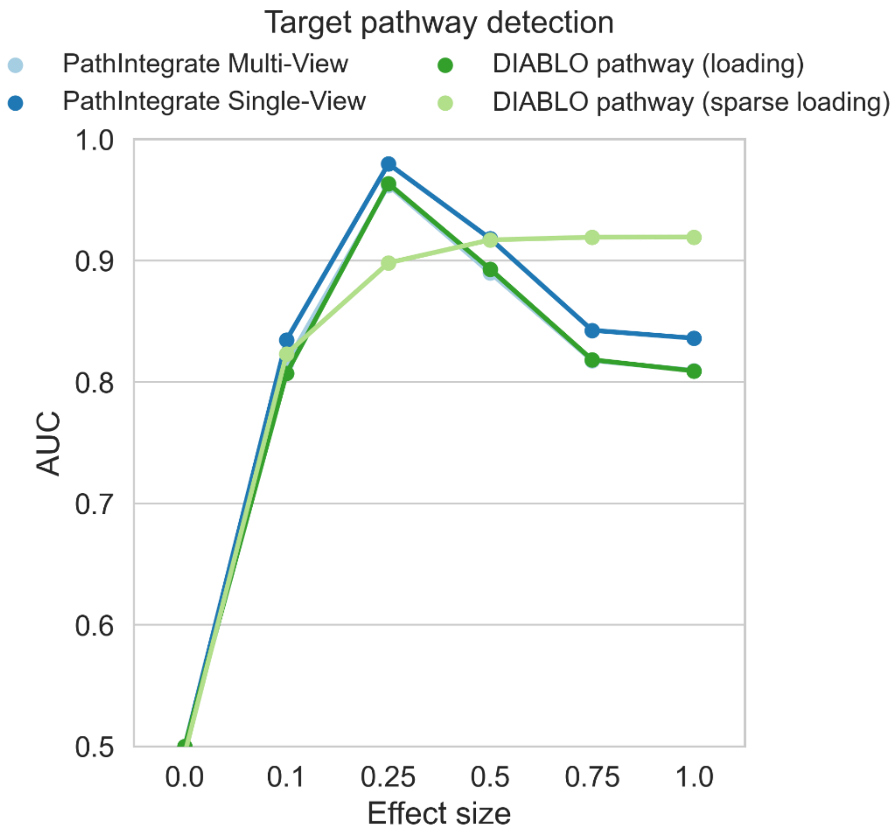

Fig F in S1 Supporting Information. *Comparison of PathIntegrate and DIABLO full/sparse models ability to correctly recall target enriched pathway based on semi-synthetic COPDGene data. 'DIABLO pathway (loading)' uses an RGCCA model with no regularisation, whereas 'DIABLO pathway (sparse loading)' uses an RGCCA model with L1 penalty.*

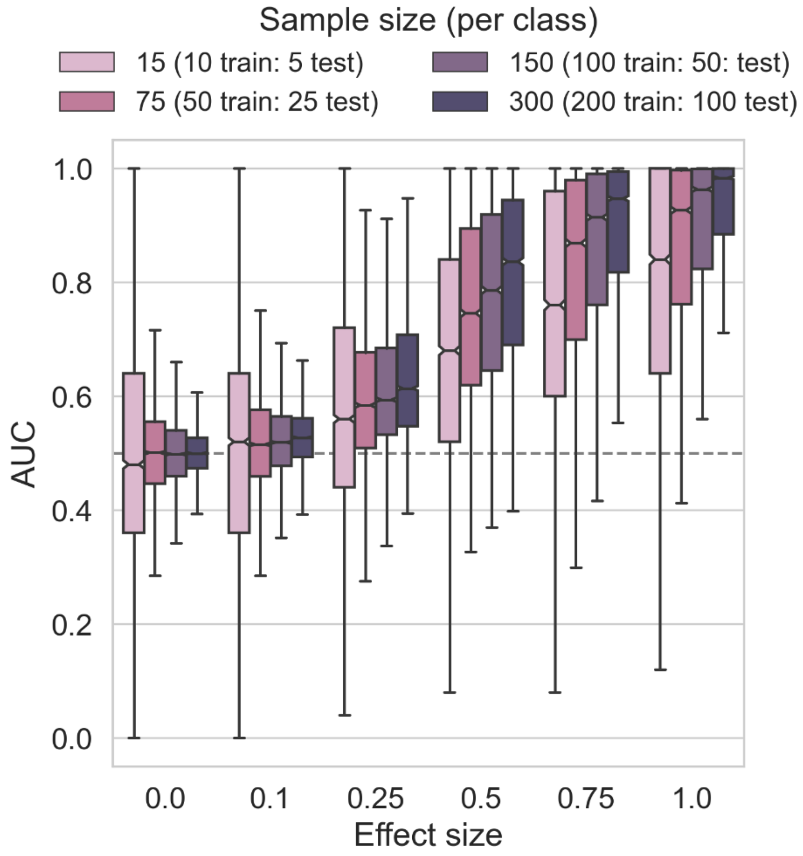

Fig *G in S1* Supporting Information*:* *Investigation of effect of sample size in PathIntegrate Single-View (PLS) classification performance on COPDgene data.*

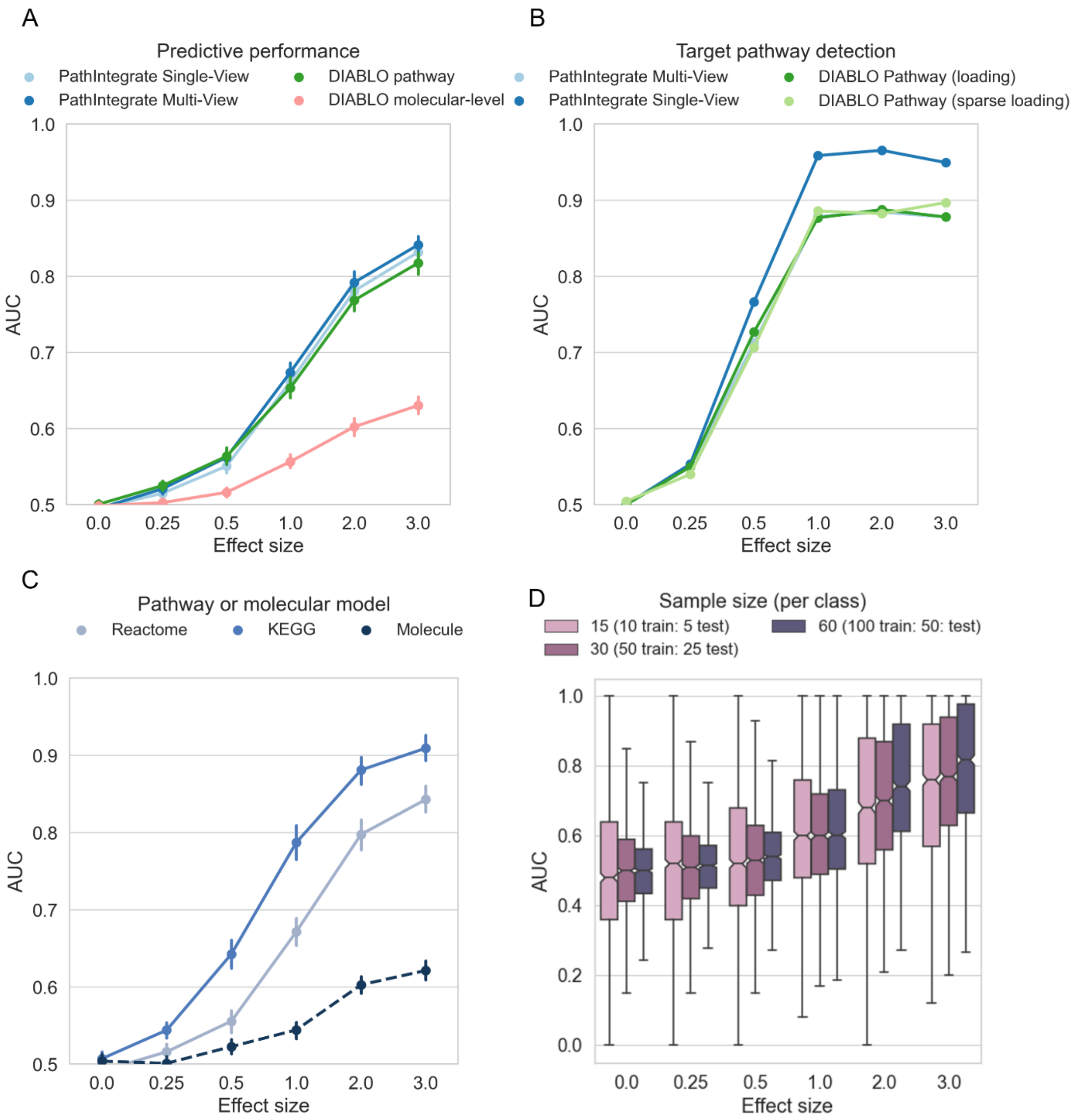

Fig *H* in *S1* Supporting Information*: Performance of PathIntegrate and DIABLO vs. effect size, based on semi-synthetic data measured by AUROC*. *COVID-19 metabolomics and proteomics data were integrated in each model. A. Ability to correctly predict sample outcomes (case vs. control). We compared PathIntegrate Multi-View and Single-View to DIABLO using both molecular and pathway-level multi-omics data. B. Ability to correctly recall target enriched pathway. For 'DIABLO pathway' we compared the full RGCCA model loadings to the sparse model loadings for feature importance. C. Comparison of PathIntegrate Multi-View using KEGG and Reactome pathway databases as well as molecular-level model. D. Effect of sample size on PathIntegrate Multi-View classification performance. For panels A-C error bars indicate 95% confidence intervals on the mean AUROC (in some cases they appear smaller than point sizes).*

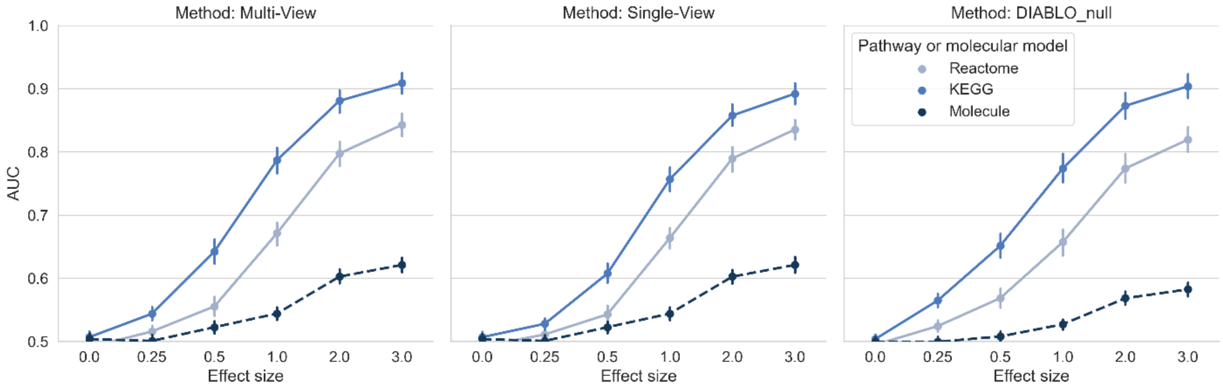

Fig I in S1 Supporting Information. *Comparison of PathIntegrate classification performance using KEGG and Reactome pathway databases as well as molecular-level model based on semi-synthetic COVID-19 data.*

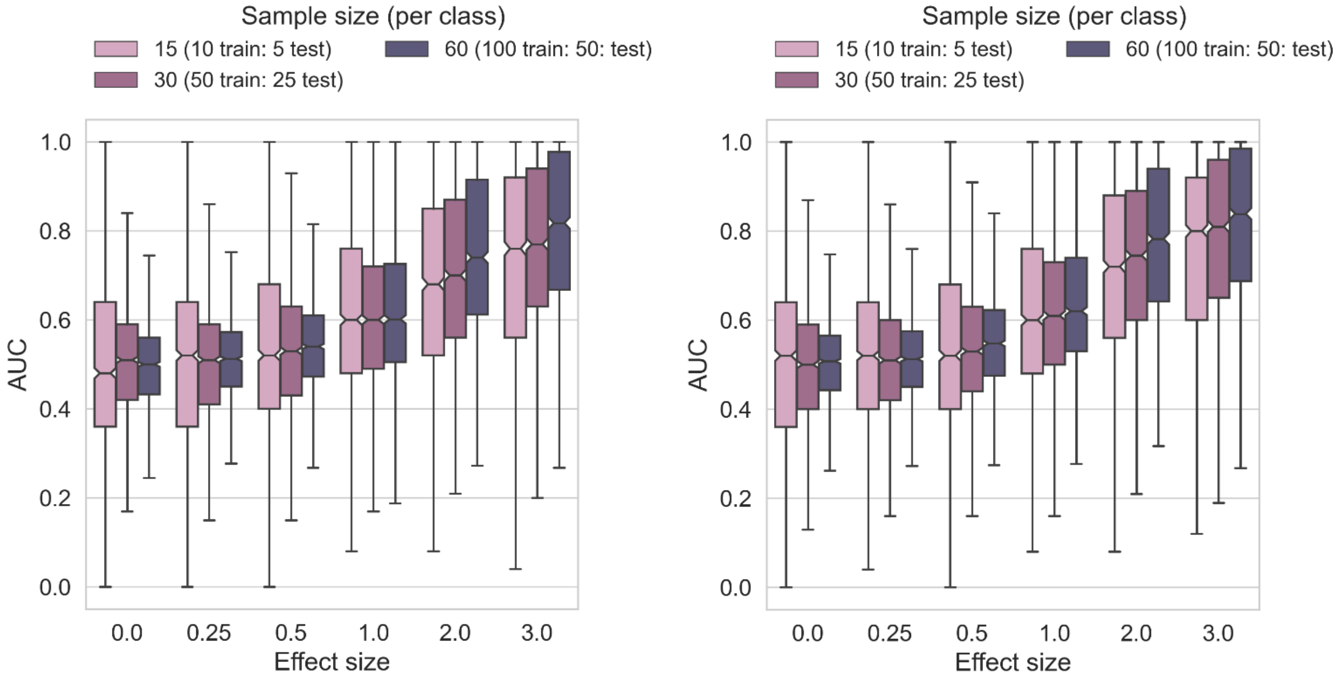

***Fig* J *in S1 Supporting Information*:** Investigation of effects of sample size in PathIntegrate Multi-View (left) and Single-View (PLS) (right) classification performance based on semi-synthetic COVID-19 data.

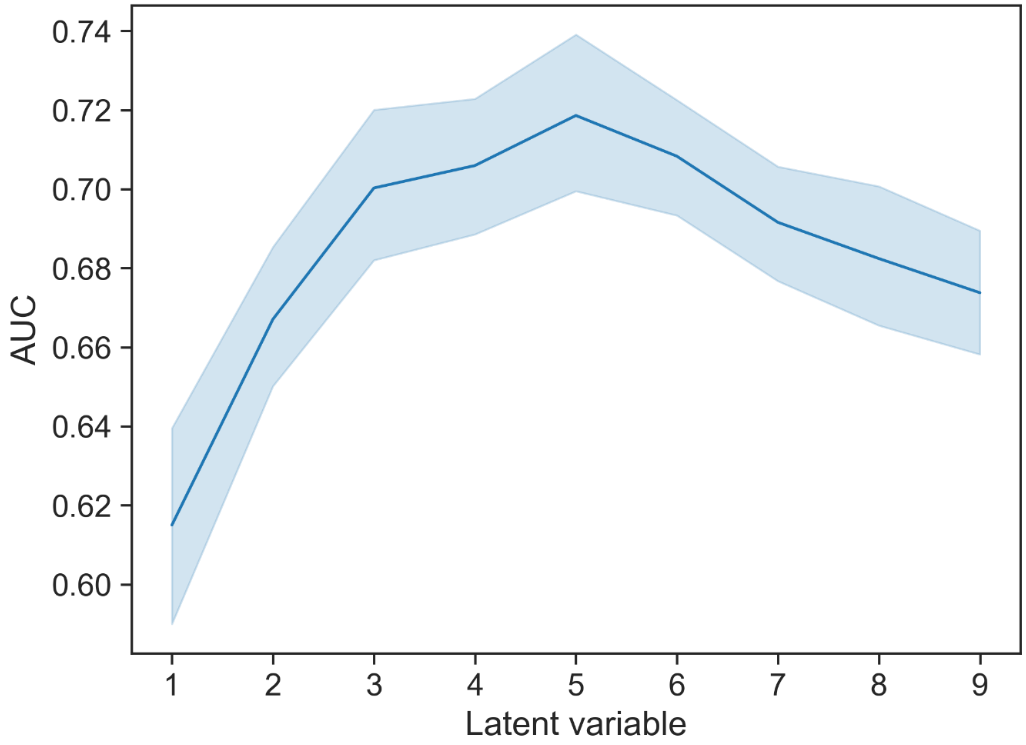

Fig K in S1 Supporting Information: *5-times repeated nested 5-fold cross-validated results for number of latent variables parameter tuning in PathIntegrate Multi-View for COPDgene case study integrating metabolomics, proteomics, and transcriptomics data. X axis shows mean AUC across inner folds. Error bars represent standard deviation.*

Table B in S1 Supporting Information: *Clinical data definitions for significantly correlated clinical variables from COPDgene study shown in Fig 4F.*

**Variable**	**Definition**
AGE_VISIT	Age in years
CAT4_Breathless	Cat questionnaire breathlessness
Finalgoldphase 2	GOLD stage at Phase 2
CurrentMedUse	Currently do you use medications to treat breathing problem
SGRQ score total	St George's Respiratory Questionnaire total score (1-100)
Predicted FEV1_FVC	Predicted ratio of the forced expiratory volume in the first one second to the forced vital capacity of the lungs
FEV1_post	Post-bronchodilator forced expiratory volume in one second
FEV1_FVC post	Post-bronchodilator forced expiratory volume in one second to the forced vital capacity of the lungs
Gender	Gender
Race of subject	Race of subject

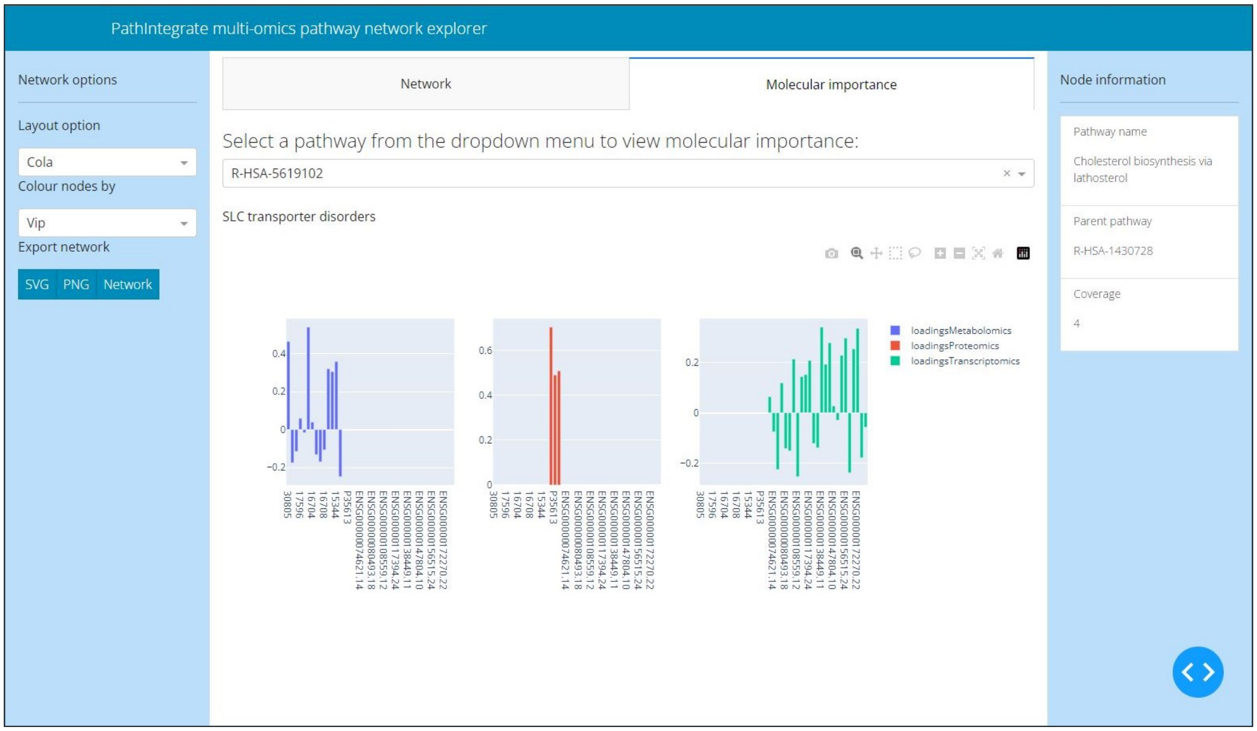

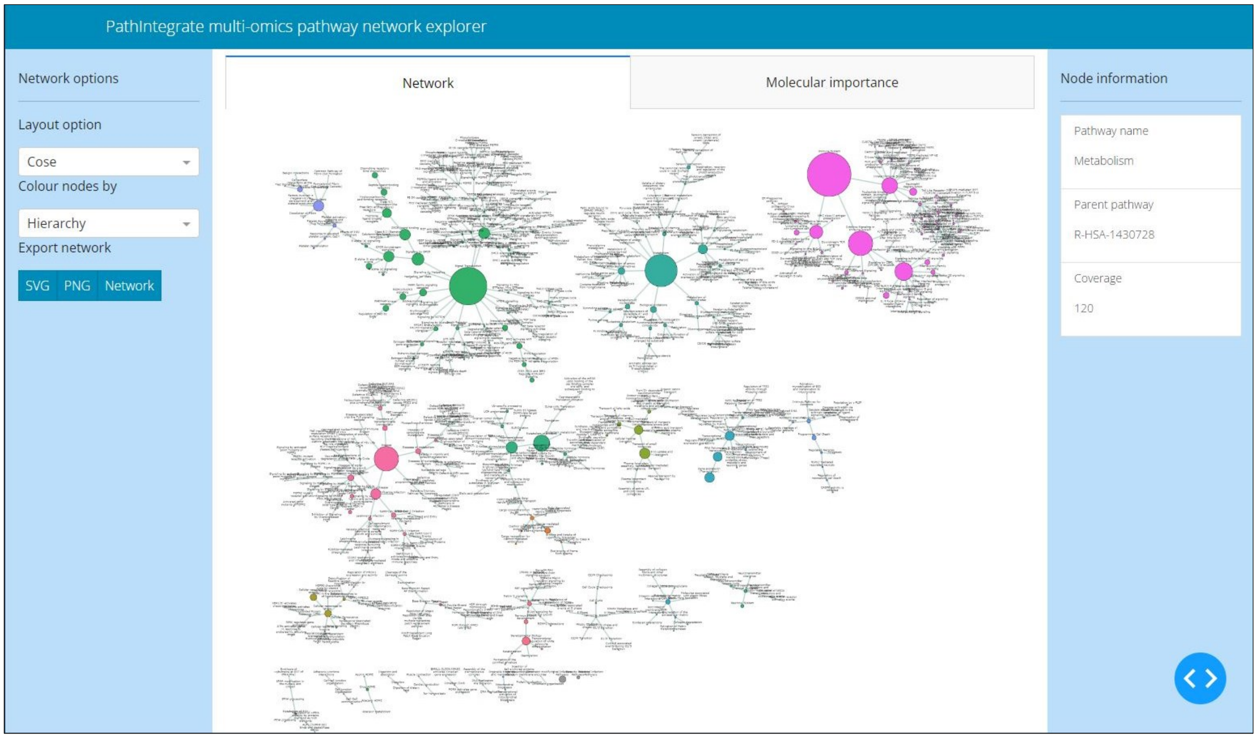

Fig L in S1 Supporting Information: *Preview of PathIntegrate network explorer app (running on a local host server) showing an example of a multi-omics dataset being analysed. Interactive visualisations are facilitated by the open-source Plotly Dash framework (MIT license). Nodes in the network represent pathways and edges represent parent-child relationships between them. Users can zoom in and hover over nodes to see more information about the pathway.*

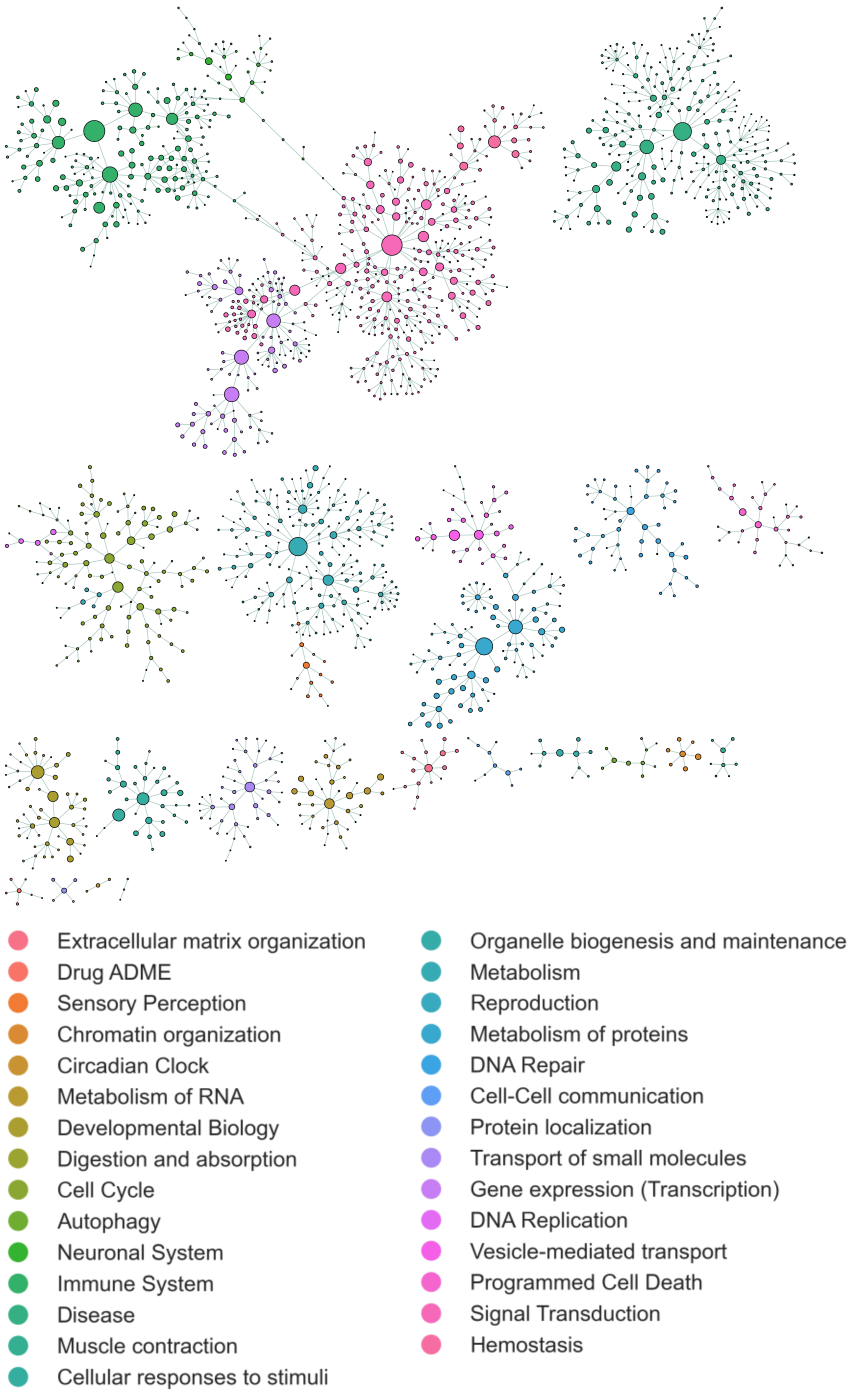

Fig M in S1 Supporting Information: *Reactome hierarchy network (based on coverage in COPDgene multi-omics data) coloured by root pathway membership with full legend. In the interactive app users can hover over nodes to see detailed information about pathway name, root pathway, and coverage in a dataset.*

Table C in S1 Supporting Information: *Table of notation*

Variable	Dimension	Definition
X	[N, M]	Molecular level matrix of N samples by M molecular features
A	[N, P]	Pathway level matrix of N samples by P pathway features
N		Number of samples profiled
M		Number of molecular features profiled
P		Number of pathways accessible in an omics dataset based on minimum coverage threshold
L		Number of molecules present in a given pathway p
p_P		A pathway member of the total pathway set P set consisting of a set of molecules
m_L		A molecule member of the pathway p_P
Z	[N, L]	Sub matrix of X containing only the L columns (molecules) present in the i'^{th} pathway
Y	[N, H]	Outcome variable
H		Number of columns in outcome variable (1 in univariate case)
$\hat{Y}$	[N, H]	Predicted outcome variable
β	[M, 1]	Set of regression coefficient of each variable in a regression model
VIP		Variable importance in projection statistic of PLS model
MB-VIP		Multi-block variable importance in projection statistic of MB-PLS model
α		Constant added to semi-synthetic data corresponding to magnitude of enrichment
C		Set of samples present in the semi-synthetic simulated control group
D		Set of samples present in the semi-synthetic simulated case group
θ		Model hyperparameters
T	[N, R]	PLS X score matrix
Ts	[N, R]	MB-PLS X super score matrix
V	[M, R]	PLS X loadings matrix (note usually denoted by P, but here we use P for pathways)
U	[N, R]	PLS Y scores matrix

C	[M, R]	PLS Y weights matrix
E, F, G	[N, M]	PLS model residual matrices
W	[M, R]	PLS X weights matrix
W^*	[M, R]	
R		Number of latent variables in PLS/MB-PLS model
k		Number of omics data matrices (predictor blocks)
J		Total number of features in a single X block
f		Total number of features across all k predictor blocks

Reference

1. Singh A, Shannon CP, Gautier B, Rohart F, Vacher M, Tebbutt SJ, et al. DIABLO: An integrative approach for identifying key molecular drivers from multi-omics assays. Bioinformatics. 2019;35: 3055–3062. doi:10.1093/bioinformatics/bty1054

2. Meng C, Basunia A, Peters B, Gholami AM, Kuster B, Culhane AC. MOGSA: Integrative single sample gene-set analysis of multiple omics data. Molecular and Cellular Proteomics. 2019;18: S153–S168. doi:10.1074/mcp.TIR118.001251

3. Jeon J, Han EY, Jung I. MOPA: An integrative multi-omics pathway analysis method for measuring omics activity. PLoS One. 2023;18: e0278272. doi:10.1371/JOURNAL.PONE.0278272

4. Hänzelmann S, Castelo R, Guinney J. GSVA: Gene set variation analysis for microarray and RNA-Seq data. BMC Bioinformatics. 2013;14: 7. doi:10.1186/1471-2105-14-7

5. Argelaguet R, Velten B, Arnol D, Dietrich S, Zenz T, Marioni JC, et al. Multi-Omics Factor Analysis—a framework for unsupervised integration of multi-omics data sets. Mol Syst Biol. 2018;14: e8124. doi:10.15252/msb.20178124

6. Al-Akwaa FM, Yunits B, Huang S, Alhajaji H, Garmire LX. Lilikoi: an R package for personalized pathway-based classification modeling using metabolomics data. Gigascience. 2018;7: 1. doi:10.1093/gigascience/giy136

7. Drier Y, Sheffer M, Domany E. Pathway-based personalized analysis of cancer. Proc Natl Acad Sci U S A. 2013;110: 6388–6393. doi:10.1073/pnas.1219651110

8. Odom GJ, Ban Y, Colaprico A, Liu L, Silva TC, Sun X, et al. PathwayPCA: an R/Bioconductor Package for Pathway Based Integrative Analysis of Multi-Omics Data. Proteomics. 2020;20: 1900409. doi:10.1002/PMIC.201900409

9. Kim SY, Jeong HH, Kim J, Moon JH, Sohn KA. Robust pathway-based multi-omics data integration using directed random walks for survival prediction in multiple cancer studies. Biol Direct. 2019;14: 1–13. doi:10.1186/S13062-019-0239-8/FIGURES/5

10. Kim SY, Choe EK, Shivakumar M, Kim D, Sohn K-A. Multi-layered network-based pathway activity inference using directed random walks: application to predicting clinical outcomes in urologic cancer. Bioinformatics. 2021;37: 2405–2413. doi:10.1093/BIOINFORMATICS/BTAB086

11. Maghsoudi Z, Nguyen H, Tavakkoli A, Nguyen T. A comprehensive survey of the approaches for pathway analysis using multi-omics data integration. Brief Bioinform. 2022 [cited 18 Oct 2022]. doi:10.1093/BIB/BBAC435
