# Supplementary material for: PathIntegrate: Multivariate modelling approaches for pathway-based multi-omics data integration
Source: PLoS Comput Biol. 2024 Mar 25;20(3):e1011814. doi: 10.1371/journal.pcbi.1011814 (PMC10994553; doi:10.1371/journal.pcbi.1011814)
Supplement: Attachment — Submitted filename: Response_to_reviews_9_02_24.docx [file pcbi.1011814.s003.docx]

Section of Bioinformatics

Division of Systems Medicine

Department of Metabolism, Digestion and Reproduction

Imperial College London

Office 315D, Burlington Danes Building

Hammersmith Hospital, Du Cane Road

London W12 0NN

Tel: +44 (0)20 7594 3160

[t.ebbels@imperial.ac.uk](mailto:t.ebbels@imperial.ac.uk)

<http://www.imperial.ac.uk/people/t.ebbels>

9 February 2024	**Prof. Tim Ebbels** BA PhD FRSC

Professor in Biomedical Data Science

Dear Editor,

We are pleased to submit a revised version of our manuscript entitled ‘PathIntegrate: Multivariate modelling approaches for pathway-based multi-omics data integration’. We thank the editors and reviewers for the time taken to consider our work, as well as the feedback and insightful comments provided. Please see below our point-by-point response to the reviewer’s comments, where new content appears in blue text and line numbers refer to those in the PDF copy of the revised manuscript. We also provide a Word copy of the revised manuscript with tracked changes.

**Reviewer #1: This is a valuable piece of work, describing a new approach for multi-omic data integration and providing interpretable and explainable predictions from these datasets through the use of pathways. The work is well described and complete. The experiments are well designed with thorough checks and good comparisons.**
 **There are a couple of limitations to this work which could be further discussed.

1. There is a nice experiment comparing the prediction with the pathintegrate approach, all molecules that are mapped to pathways and finally prediction using all molecules whether mapped or not. I would appreciate additional discussion around the effect of having unmapped molecules, what is the potential impact on the prediction, and also how does this limit the discovery of new biology through this type of anlaysis.**

We thank the reviewer for raising this important point. We have added some discussion on this as follows:

L589-596: In the COPDgene case study, we compared the predictive performance of the PathIntegrate Multi-View model to its molecular level counterpart, including molecules not annotated to pathways. We did not find including un-annotated molecules lead to significant changes in performance in this case, however the reliance on current knowledge is a widely accepted limitation of pathway-based methods, which can be expected to improve as annotations become more complete. Meanwhile, future work should focus on how un-annotated molecules could be incorporated into pathway-based models, to minimise information loss. **2. When discussing multi-omics and missing data, you might want to look into MOFA which can handle missing data rather well in its models.**

We thank the reviewer for highlighting this and have referred to MOFA and other probabilistic approaches in relation to handling missing values in the discussion:

## L658-666: In common with many other statistical integration approaches, PathIntegrate requires all input omics to be measured on the same individuals. This means samples from individuals without data on all omics will have to be discarded, as PathIntegrate currently does not support entire rows of missing data. Some models can accommodate sparse data where values are missing at random 68, including MB-PLS (using NIPALS algorithm 69) and probabilistic frameworks such as MOFA 13. However, further work is required to develop multi-omics integration methods that can handle samples with one or more omics missing 70 (missing not at random 68). Additionally, using pathway-based models may aid in the robust imputation of data, by helping to capture biological rather than technical variation.

**Reviewer #2: This manuscript proposes a novel framework (PathIntegrate) for integrating multiple blocks/types of omics data. This framework first reduces the data from the molecular to the pathway level before applying MB-multivariate or classification modelling. The Authors report advantages in signal detection (i.e. elucidating information from low effect sizes) and improved interpretability of results. Overall, the research is technically sound and I recommend that it be published. The comments below are suggested improvements and queries.**

**1. Figure 2 presents a nice visual summary of the framework after the pathway transformation step is complete. It would be helpful for the readers to see this earlier (at the beginning of the Results) and have the pathway transformation step included. That way, the readers can see the whole framework before they start reading about the details.**

We agree with the reviewer that a visual summary of the pathway transformation step is important, especially for readers without this background. As such we have added a new Figure 1 to the introduction where pathway transformation/ssPA is first mentioned, to provide an overview of the concept. This way, we hope that when readers view Figure 3 (the PathIntegrate modelling frameworks overview), it will be clear what the pathway transformation step is referring to.  **2. The link to the tutorials and documentation doesn't work (the link appears to be case sensitive). This direct link may not be needed as it is easy to find the tutorials and documentation through the GitHub page. I commend the authors for including a Google Colab tutorial - it is easy to follow.**

We thank the reviewer for looking into this and have fixed the link to the tutorials. We are glad the reviewer found the Colab tutorial easy to follow and will plan to update these periodically based on user feedback and new developments.  **3. Lines 105-107, the Authors state that one of the challenges of multi-omics integration is the heterogeneity in the number of features profiled. They go on to say that PathIntegrate addresses this challenge in the pathway transformation step. However, the pathway coverage of the different omics blocks will still exhibit heterogeneity (this is partially observed in Figure 4D). Can the Authors please elaborate on how PathIntegrate resolves this challenge?**

We thank the reviewer for highlighting this important point and agree that PathIntegrate cannot fully resolve this. While it brings different omics to a common scale (i.e. in terms of pathway activity values), it cannot guarantee the number of pathways in each omics block will be very similar, as this is highly dependent on the pathway resource used as well as biological considerations. We have changed the wording in the introduction to reflect this:

L107-109: PathIntegrate substantially **contributes to addressing** these within the pathway-transformation step, where disparate omics datasets are brought to a common scale, i.e. in terms of pathway ‘activity’.

And have also added further discussion about this:

L600-605: While the pathway transformation step in PathIntegrate can result in transformed omics datasets having more homogenous numbers of pathway features, this is not always the case, and depends on a several factors including the pathway resource used, the coverage of the assay technology, as well as the underlying biology (e.g. there are inherently fewer known metabolic pathways than signalling pathways).

**4. I observed some Methods content in the Results (example paragraph starting at line 153) and some Discussion content in the Results (examples: lines 173, 282, 340). The Authors may wish to review content placement for improved cohesion.**

We thank the reviewer for this suggestion. While we agree that the paragraph starting at line 153 (160 in the revised manuscript) contains several methodological details, we feel that these are necessary to aid understanding of the simulation performed and the results presented, as this is a non-standard analysis and simulation procedure being introduced for the first time in the paper.

The content beginning at lines 173, 282, and 340 (180, 289, 347 in the revised manuscript) is designed to summarise the results of each subsection and serve as a transition to the next section, which we believe facilitates comprehension. For this reason, we feel it is important to retain this content where it is, however if the editor feels strongly, we are open to changing it.

All authors have approved the revised manuscript and agree with its submission to PLOS Computational Biology. Please address all correspondence to [t.ebbels@imperial.ac.uk](mailto:t.ebbels@imperial.ac.uk)

Yours sincerely,

Timothy Ebbels

Imperial College London
